# Comparison of 3D-Printed Dental Implants with Threaded Implants for Osseointegration: An Experimental Pilot Study

**DOI:** 10.3390/ma13214815

**Published:** 2020-10-28

**Authors:** Ling Li, Jungwon Lee, Heithem Ben Amara, Jun-Beom Lee, Ki-Sun Lee, Sang-Wan Shin, Yong-Moo Lee, Byoungkook Kim, Pangyu Kim, Ki-Tae Koo

**Affiliations:** 1 Department of Periodontology and Dental Research Institute, School of Dentistry, Seoul National University, Seoul 03080, Korea; applemint1228@snu.ac.kr (L.L.); benamarahaitham@yahoo.fr (H.B.A.); dentjblee@gmail.com (J.-B.L.); ymlee@snu.ac.kr (Y.-M.L.); 2 One-Stop Specialty Center, Seoul National University Dental Hospital & Department of Periodontology, School of Dentistry, Seoul National University, Seoul 03080, Korea; jungwonlee.snudh@gmail.com; 3 Department of Prosthodontics, Korea University Ansan Hospital, Ansan 15355, Gyeonggi-do, Korea; kisuns@gmail.com; 4 Department of Advanced Prosthodontics, Graduate School of Clinical Dentistry, Korea University, Seoul 02841, Korea; swshin@korea.ac.kr; 5 3D Printer R&D Team, Dentium Co., Ltd., Suwon 16229, Gyeonggi-do, Korea; bkkim1@dentium.com (B.K.); pgkim@dentium.com (P.K.)

**Keywords:** 3D printing, computer-aided design, customized dental implant, patient matched, implant stability

## Abstract

This study aimed to compare bone healing and implant stability for three types of dental implants: a threaded implant, a three-dimensional (3D)-printed implant without spikes, and a 3D-printed implant with spikes. In four beagle dogs, left and right mandibular premolars (2nd, 3rd, and 4th) and 1st molars were removed. Twelve weeks later, three types of titanium implants (threaded implant, 3D-printed implant without spikes, and 3D-printed implant with spikes) were randomly inserted into the edentulous ridges of each dog. Implant stability measurements and radiographic recordings were taken every two weeks following implant placement. Twelve weeks after implant surgery, the dogs were sacrificed and bone-to-implant contact (BIC) and bone area fraction occupied (BAFO) were compared between groups. At implant surgery, the primary stability was lower for the 3D-printed implant with spikes (74.05 ± 5.61) than for the threaded implant (83.71 ± 2.90) (*p* = 0.005). Afterwards, no significant difference in implants’ stability was observed between groups up to post-surgery week 12. Histomorphometrical analysis did not reveal a significant difference between the three implants for BIC (*p* = 0.101) or BAFO (*p* = 0.288). Within the limits of this study, 3D-printed implants without spikes and threaded implants showed comparable implant stability measurements, BIC, and BAFO.

## 1. Introduction

Various types of dental implants have been developed to replace edentulous areas. Endosseous blade implants and disk implants designed in the 1960s disappeared from the market because of their low survival rate and the extensive bone destruction that would occur around the implant [1,2]. Endosseous dental implants have been considered as the current standard shape, and the surface in contact with the bone is subjected to large shear forces under load [3].

The most widely used implants on the market today are threaded implants. However, such implants are limited in design owing to the need for mass production. Close contact between the recipient site and the implant is essential for proper osseointegration [4]. Therefore, the current implants cannot completely satisfy the requirements of individual patients [5]. Titanium implants with high surface porosity and high core density may allow better load adaptation, while avoiding stress shielding and pressure-induced bone loss [6,7,8]. However, the manufacturing of personalized implants with this structure is considerably challenging and expensive. In contrast, if a three-dimensional (3D) printing method is used, personalized implants with a complex structure can be manufactured at a lower cost and with more simplicity [9].

Recently, the application of 3D printing technology in dentistry has grown at a rapid pace. The reasons for this rapid development are the possibility of savings on small-scale productions, the ability to manufacture personalized products, the ease of sharing and handling patient imaging data, and the increased number of people who understand and can carry out this process [10]. 3D printing technology is gaining increased attention in the dentistry field thanks to advances in 3D imaging and modeling technologies, such as intraoral scanning and cone-beam computed tomography, and the increasing use of computer-aided design & computer-aided manufacturing (CAD/CAM) technology [11]. 3D printing is also known as additive manufacturing, in which rapid prototyping uses a focused laser beam to create complex shapes layer-by-layer [12]. Implants made using 3D printing technology are custom designed to accommodate the geometry of each individual’s anatomic structure, preserving soft and hard tissue and reducing the duration of the healing period [5,13]. The technology makes it easier to create an implant with complex structures. In addition, unlike the cutting or milling process, 3D printing can be conducted without molds or other tools, which is more economical and reduces material loss [14].

Although 3D printing technology is used in many aspects of the dental field, clinical data are still limited regarding the manufacturing of dental implants. The purpose of this study was to compare the sequential implant stability and histologic differences between 3D-printed implants and threaded implants.

## 2. Materials and Methods

### 2.1. Animals

**Figure 1:**
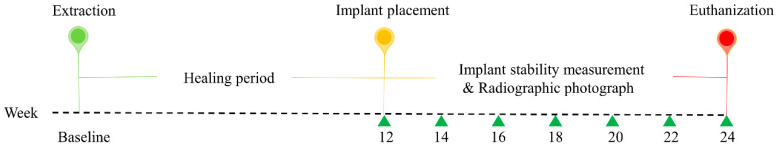
Outline of the experiment. At baseline, the second, third, and fourth premolars and first molars were extracted from the left and right sides of the mandible. After 12 weeks, three different implants were randomly placed in the healing ridges.

The study protocol was approved by the Institutional Animal Care and Use Committee (IACUC), Seoul National University (IACUC No. SNU-190226-4), and the study was conducted in compliance with guidelines of the Institute of Laboratory Animal Resources, Seoul National University. This research regulated by the principle of the 3Rs (replacement, reduction, and refinement). Four male beagle dogs, at 1 year of age and a weight between 10 and 12 kg, were used in the study. At the time of recruitment, all animals were healthy and the dentition was normal. Before the experiment, the beagle dogs were acclimated to the facility for 1 week. The dogs were individually housed in 90 cm × 80 cm × 80 cm (width × depth × height) indoor kennels. They drank freely and were fed a standard pellet dog food diet (HappyRang, Seoulfeed, Korea) or a crushed diet after the implants were placed. The study outline is presented in Figure 1.

### 2.2. Study Implants

**Figure 2:**
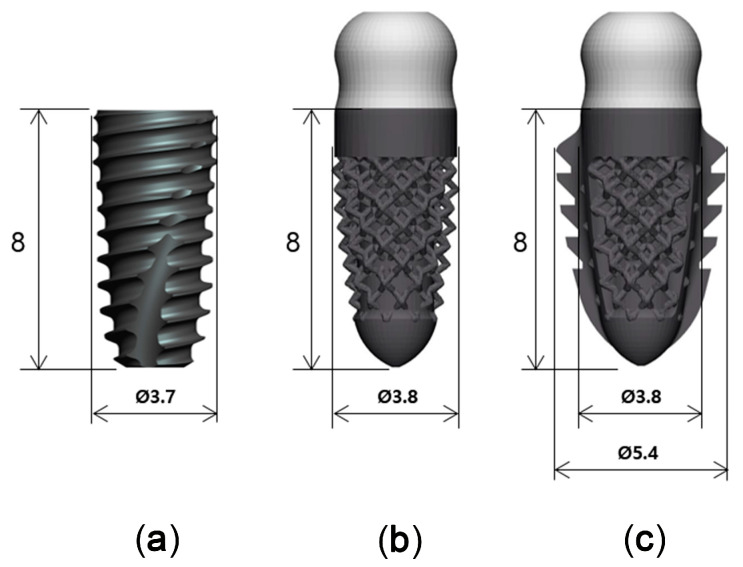
Three implants with different designs. (**a**): SuperLine Fixture (FXS 36 08); (**b**): 3D-printed implant without spikes; (**c**): 3D-printed implant with spikes.

Titanium implants with three different designs, but with similar dimensions, were used in this study (Figure 2): a threaded implant (Superline, Dentium, Seoul, Korea), which was a two-piece bone level implant with 3.7 mm in diameter and 8 mm in length (T; control); a 3D-printed implant without spikes, which was a one-piece tissue level implant with 3.8 mm in diameter and 8 mm in length (3D; test 1); and a 3D-printed implant with spikes, which was a one-piece tissue level implant with 3.8 mm in diameter and 8 mm in length (3DS; test 2).

**Figure 3:**
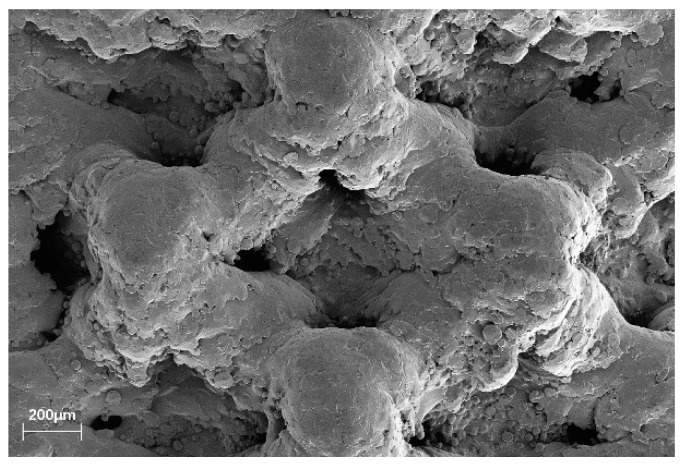
Scanning electron microscopy (SEM) image of the 3D-printed implant without spikes macroporous structure. Both 3D-printed implants feature the same surface.

The 3D-printed implant used a dodecahedron lattice structure with a porosity of 70% and a lattice thickness of 250 μm (Figure 3). They were designed with and without spikes to compare the influence of the spikes on implant stability. The spikes’ structure was designed in order to provide additional fixation on the extraction site and sidewalls. The 3D-printed implants were printed on a 3D printer Mlab200R (Concept Laser, Lichtenfels, Germany) using Ti-Gr2 (ConceptLaser, Lichtenfels, Germany). The 3D printer uses direct metal laser melting (DMLM) technology to create complex metal 3D geometries. For the surface of the 3D-printed implant, only blasting was performed without any surface treatment. The T implant encompasses sandblasting with large grits and acid etching (SLA) surface and is made of Ti-Gr5.

In order to identify the precision of the 3D-printed products, five rectangular parallelepipedons having a width of 15 mm, a length of 15 mm, and a height of 6 mm were manufactured. When measuring the length of the manufactured rectangular parallelepiped, the maximum error was 9.7 µm (Table 1).

### 2.3. Surgical Procedure

All surgical procedures were performed under general anesthesia with an intravenous injection of Zoletil^®^ (5 mg/kg; Virbac, Carros, France), Rompun^®^ (2.3 mg/kg; Bayer Korea, Ansan, Korea), and atropine sulfate (0.05 mg/kg; Jeil Pharm., Daegu, Korea). A complementary local anesthesia was performed at surgical sites with 2% lidocaine HCL with epinephrine (1:1,000,000) (20 mg/kg; Huons, Seongnam, Korea). On the left and right side of the mandible in all dogs, the second, third, and fourth premolars (PM2, PM3, and PM4, respectively) and the first molars (M1) were atraumatically extracted with flap elevation. To reduce damage to the alveolar bone, a hemisection was made in the buccolingual direction of the teeth with diamond fissure burs. The surgical site was closed with a suture (Monosyn 5/0, B Braun Aesculap, Tuttlingen, Germany), and the suture was removed after 1 week.

**Figure 4:**
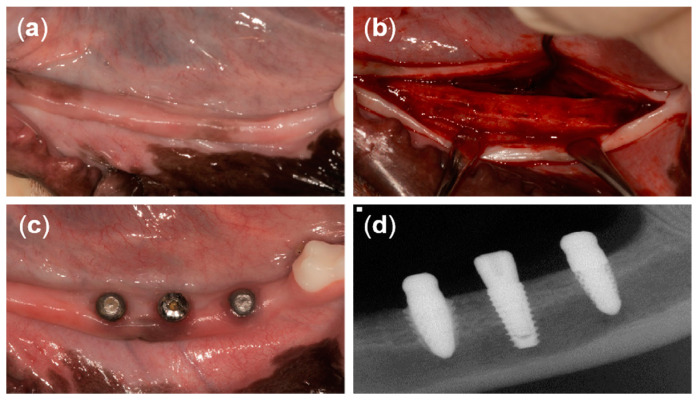
Clinical photographs from the present study. (**a**) Twelve weeks after tooth extraction, (**b**) horizontal incision and flap reflection, (**c**) 8 weeks after implant placement, and (**d**) periapical radiograph of three different types of implants after 8 weeks of healing.

At 12 weeks after tooth extraction, an incision was made in the crest to place the implants (Figure 4a,b). A full-thickness flap was reflected and drilled for the placement of three different implants. The drilling sequence for the T implant was accomplished following the manufacturer’s recommendations. The drilling sequence for the 3D implant started from the initial drill (2.2 mm diameter), a second drill (2.6 mm diameter), two twist drills (2.9 mm and 3.35 mm in diameter), and then ended with the countersink drill (3.6 mm diameter). The drilling sequence for the 3DS implant started from the initial drill (2.2 mm diameter), a second drill (2.6 mm diameter), three twist drills (2.9 mm, 3.35 mm, and 3.85 mm in diameter), and then ended with the countersink drill (4.5 mm diameter). The implants were randomly placed at three positions on each of the left side and the right side of the mandible (six implants per dog). A random sequence was generated using the simple method of the www.random.org website. T implants were placed using a motor-driven handpiece (EXPERTsurg™ LUX, KaVo, Germany) and 3D and 3DS implants heads were directly tapped using a surgical mallet. Subsequently, the T implants were secured with a cover screw. The flap was repositioned and sutured with 5/0 Monosyn^®^ (B Braun Aesculap, Tuttlingen, Germany), and the sutures were removed 1 week later. The surgical procedures were performed by one periodontist.

### 2.4. Postoperative Management

To mitigate postoperative pain and inflammation, antibiotics (Cefazoline 20 mg/kg; Chongkundang Pharm., Seoul, Korea), analgesics (Toranzin 5 mg/kg; Samsung Pharm., Gyeonggi-do, Korea), and antispasmodics (atropine sulfate 0.05 mg/kg; Jeil Pharm., Daegu, Korea) were intravenously injected after tooth extraction and implant placement. In addition, antibiotics (amoxicillin 500 mg; Chongkundang Pharm., Seoul, Korea) and analgesics (ibuprofen 400 mg, Daewoong Pharm., Seoul, Korea) were mixed with the animals’ diet three days after the operations. All dogs were placed under soft diet for one month after surgery to avoid any mechanical interference with the postsurgical healing, and surgical areas were checked twice per week to confirm whether any complications had occurred. Meanwhile, all the surgical sites were rinsed with 0.12% chlorhexidine gluconate solution after feeding (Hexamedine^®^, Bukwang Pharm., Seoul, Korea).

### 2.5. Implant Stability Measurements 

As a result of limitations of resonance frequency analysis in measuring the 3D-printed implants’ stability, damping capacity analysis (Anycheck, Neobiotech, Seoul, Korea) was used for implant stability analysis [15]. The implant stability measurements and periapical radiographic recordings were performed every two weeks following implant placement under general anesthesia. Each implant was measured five times on the buccal side. The average value of the measurements from the five times was considered as representative for each implant.

### 2.6. Histologic Observation and Histomorphometric Analysis

**Figure 5:**
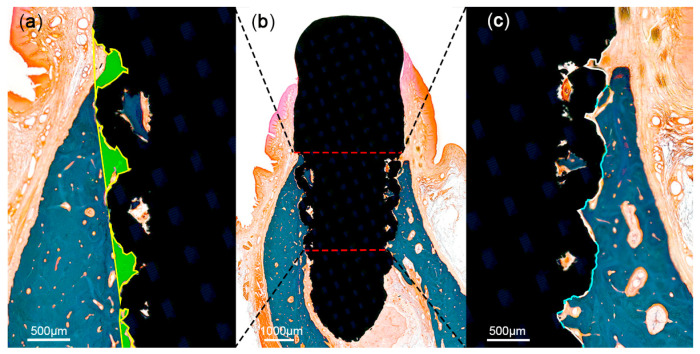
Schematic illustration of histomorphometric analysis in the region of interest (ROI). The ROI was set at the coronal half of each implant (**b**). Areas of the mineralized bone (green) were defined within the implant (**a**). Tissue-to-implant contact within the ROI (**c**) was differentiated into mineralized bone (blue) and void (white).

At 12 weeks after implant placement, the beagle dogs were sacrificed by carotid injection with potassium chloride (75 mg/kg; Jeil Pharm., Daegu, Korea). Block biopsies including the extraction sites were collected for histologic observation and histomorphometric analysis. The specimens were placed in a fixative solution containing 10% neutral formalin buffer for 1 week, and subsequently dehydrated in graded ethanol solution. Thereafter, the samples were embedded in resin blocks (Technovit 7200; Heraeus Kulzer, Hanau, Germany) with a UV embedding system (KULZER EXAKT 520, Hanau, Germany) according to the manufacturer’s recommendation. The sectioning procedure was performed using a diamond saw. Thereafter, the sections were ground and polished to approximately 80 ± 5 μm, and then stained with Goldner trichrome. On slide scans produced at 20× magnification, histomorphometric analysis was performed using ImageJ 1.51j8 (National Institutes of Health, Bethesda, MD, USA) to measure bone-to-implant contact (BIC) and bone area fraction occupied (BAFO) in a region of interest (ROI) set to the coronal half of each implant (Figure 5).

### 2.7. Statistical Analyses

The sample size of eight per group was obtained by power analysis under the assumption of a mean difference of 20 among a control group and two experimental groups, with a common sd of 12 (effect size: Cohen’s f = 0.786) and 90.0% of power, 0.05 alpha level using GPower version 3.1.9.2. 

Statistical analyses were performed using SPSS version 25 (IBM Software, Armonk, NY, USA). Descriptive statistics are expressed as the mean ± standard deviation. The Kruskal–Wallis test was used to compare the effect of different implant designs with the results of implant stability values and histomorphometric data (BIC and BAFO) at an alpha level of 0.05. In the case of statistically significant differences, pairwise post hoc comparisons were performed at *p* = 0.017 significance level using the Mann–Whitney test under the Bonferroni-corrected significance level. The values tested every two weeks were used to observe the correlation of the implant stability values between the three different implants.

## 3. Results

### 3.1. Clinical and Radiographic Observations

The healing of the implants was uneventful, with no clinical signs of inflammation except at two PM2 sites—a T implant placed on the right side and a 3D implant placed on the left side.

### 3.2. Implant Stability Measurements

Except for two implants, the stability values for all other implants ranged from 71.18 ± 5.06 to 82.63 ± 4.09 (Table 2). There were significant differences between the three implant types at the time of surgery (*p* = 0.007) and at 2 weeks post-surgery (*p* = 0.048). The pairwise post hoc comparisons showed that the initial fixation force was lower for 3DS implant than for T implant (*p* = 0.005), but no difference was observed after 2 weeks. In addition, no significant differences in mean implant stability were observed from weeks 4 to 12 (Table 2). In 3D and 3DS groups, the implant stability values decreased at the 2-week observation compared with the time of surgery; however, the values gradually increased from week 4 to week 12. In the T group, the implant stability values decreased until 4 weeks post-surgery, but then gradually increased from week 6 to week 12 (Figure 5).

### 3.3. Histologic Observations

**Figure 6:**
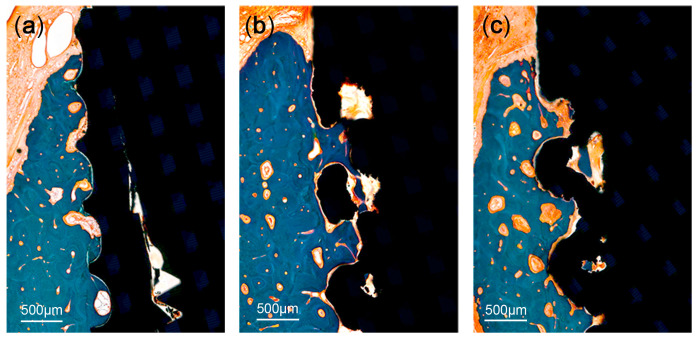
Histologic photograph of threaded implant: (**a**) 3D-printed implant without spikes, (**b**) 3D-printed implant with spikes, and (**c**) at 12 weeks following implant placement. The region within threads and within lattices was occupied with new bone. Primary bone remodeling had nearly ceased, while secondary remodeling was ongoing around all types of implants.

A significant bone loss was found in the implants placed in the PM2 sites (a T implant and a 3D implant). These two implants were not included in the measurements. There was no evidence of inflammatory response in any specimen examined, except for the two implants. The coronal area of the implant showed more bone-to-implant contact, while the apical area showed relatively more contact between bone marrow and the surface of the implants. The region within threads and within lattices were occupied with new bone. Primary bone remodeling had nearly ceased, while secondary remodeling was ongoing around all types of implants (Figure 6).

### 3.4. Histomorphometric Analysis

Mean values (± standard deviation) of BIC and BAFO are presented in Table 3. With regard to BIC, the T implant, the 3D implant, and the 3DS implant averaged 52.27 ± 13.78%, 59.43 ± 16.98%, and 44.28 ± 15.99%, respectively. There were no significant differences in the BICs between the three groups (*p* = 0.101). The mean BAFO was 56.79 ± 11.25%, 56.98 ± 12.48%, and 45.58 ± 10.77% in T group, in 3D group, and 3DS group, respectively. No significant differences were observed between the three groups (*p* = 0.288).

## 4. Discussion

In the present study, 3D-printed implants and conventional threaded implants were compared through stability measurements and histological analysis. Overall, comparable results were found in terms of implant stability, BIC, and BAFO irrespective of the studied implant. However, in this study, the two-dimensional histological assessment of the bone–implant interface was performed. This should be complemented by a 3D evaluation allowing a more accurate comparative analysis.

The lattice structure in the 3D-printed implants (3D and 3DS) did not appear to affect their stability in bone (Table 2). Hence, the 3D implant achieved and preserved primary stability similar to the T implant. A significant difference was found between the primary stability of the 3DS implant and T implant. This might be explained by the surgical preparation of the 3DS implant bed requiring a larger osteotomy for the spikes. In turn, a gap was created between the bony walls of the surgical bed and the surface of the 3DS implant, translating into lower implant stability measures. These values, however, remain within the favorable range for primary stability [16]. Thereafter, the stability values described a trend toward a decrease followed by an increase over time. This observation is related to the discrepancy that exists between the rate of primary stability decrease and the rise of secondary stability throughout the healing process [17,18], as reported with threaded implants [19,20,21]. The outcomes from the previous studies, obtained using the resonance frequency analysis, are in line with the results herein, although the latter were produced with the damping capacity analysis [15,16,17,18,19,20,21,22].

Several studies have previously reported the outcomes of 3D-printed titanium implants in pre-clinical and clinical settings. Stubinger et al. [23] used the sheep pelvis model to analyze the in vivo characteristics of implants made by direct metal laser sintering. The implants made by this technology had a porous structure, and when compared with controls with standard machined, sandblasted, and etched surfaces after 2 and 8 weeks, the direct metal laser sintering implant did not show significant differences in BIC as compared with the other implants. Between the two observation time points, the direct metal laser sintering implant showed the highest increase in BIC. Compared with the machined implant and sandblasted and etched implant, the removal torque test of the direct metal laser sintering implant surface showed a significant improvement in the fixed strength after 8 weeks. In the study by Witek et al. [24] comparing a Ti-6Al-4V threaded type implant made by laser sintering to a control group with alumina blast and acid-etched surface after 1, 3, and 6 weeks, the BIC and BAFO values of the laser-sintered implant were higher at 1 week compared with the control group and did not show a significant difference at 3 and 6 weeks. In addition, after 1 and 6 weeks, the laser sintering implant showed a significantly higher removal torque value. Likewise, in a 1-year follow-up of 3D-printed custom-made implants in humans, no impairment of stability or signs of infection were observed simultaneously with a complete function and aesthetic integration [6,7,8,9,10,11,12,13,14,15,16,17,18,19,20,21,22,23,24,25]. Another 3-year follow-up clinical trial reported a survival rate of 94.5% and a crown success rate of 94.3% with 3D-printed implants [26]. Although the direct comparison of the previous studies might be questioned with regard to the different experimental protocols and implants used, the reported results overall indicate that the bone around implants made by 3D-printing displays favorable remodeling features and biomechanical stability. It can also be seen that the 3D printing manufacturing process does not adversely modify the biological or chemical properties of the material.

The microscopic factors and macroscopic factors of the implant are essential factors for implant stability and biological response [27]. To stimulate the growth of new bone into the pores, a materials’ porosity superior to 60% is required [28]. This porosity can lead to interconnected porous structures, which facilitates cell ingrowth into porous spaces and facilitates vascularization and metabolite transport [28]. The three-dimensional lattice structure was used to increase the surface area of the 3D-printed implants. Therefore, the lattice structure surface with a porosity of 70% used in this study promotes the growth of new bone into pores to increase bone fixation. To mitigate bacterial colonization around the implant–bone interface, a lattice structure was not used at the top 1.5 mm of the implant texture. The implant with the SLA surface has better cell adhesion and bone neoformation than the machined surface [29]. In this study, 3D-printed implants with untreated surface and threaded implants with SLA surface were compared, and there was no statistically significant difference in histological and biomechanical properties. It is the lack of this research that brings micro and macro factors into the equation. In a follow-up study, SLA surface treatment will be performed on the 3D-printed implant to compensate for the limitation of this pilot study. Several animal models have been used for evaluating the biocompatibility of implants, but the canine model is known to be the most appropriate for implant material testing thanks to its close similarity in bone composition to humans [30,31]. A small amount of research has been conducted thus far on 3D-printed dental implants. In this perspective, the present study provides useful data for the characterization of bone healing at the surface of 3D-printed implants.

## 5. Conclusions

Within the limits of this study, both types of 3D-printed implants tested in the present study (3D and 3DS) showed comparable implant stability as well as BIC and BAFO values with T implants up to 12 weeks following insertion.

## Figures and Tables

**Table 1:** Accuracy analysis of 3D-printed products.

Test	x (mm)			y (mm)			z (mm)		
	Reference Values	Measures	Error	Reference Values	Measures	Error	Reference Values	Measures	Error
1	15.0000	15.0097	0.0097	15.0000	15.0045	0.0045	6.0000	6.0034	0.0034
2		15.0081	0.0081		15.0067	0.0067		6.0029	0.0029
3		15.0061	0.0061		15.0018	0.0018		6.0011	0.0011
4		15.0042	0.0042		15.0061	0.0061		6.0005	0.0005
5		15.0084	0.0084		15.0052	0.0052		6.0044	0.0044
Mean		15.0073	0.0073		15.0049	00049		6.0025	0.0025

**Table 2:** Comparison of implant stability measurements among the three different implants.

Time	Threaded Implant (N = 7)	3D-Printed Implant without Spikes (N = 7)	3D-Printed Implant with Spikes (N = 8)	*p*-Value
Surgery	83.71 ± 2.90 ^a)^	79.49 ± 3.94 ^a),b)^	74.05 ± 5.61 ^b)^	0.007
2 weeks	76.29 ± 2.90	77.06 ± 2.90	71.18 ± 5.06	0.048
4 weeks	75.23 ± 3.22	77.80 ± 2.45	72.28 ± 5.52	0.112
6 weeks	75.86 ± 3.95	78.89 ± 1.37	76.00 ± 4.61	0.221
8 weeks	75.37 ± 5.29	77.89 ± 1.86	76.98 ± 3.57	0.815
10 weeks	76.11 ± 3.79	78.80 ± 1.89	77.75 ± 3.50	0.319
12 weeks	77.00 ± 4.30	80.17 ± 2.97	79.45 ± 2.74	0.349

Values are presented as the mean ± standard deviation. *p*-values were calculated using the Kruskal–Wallis test to compare implant stability values among the threaded implant, 3D-printed implant without spikes, and 3D-printed implant with spikes (*p* < 0.05). Pairwise post hoc comparisons were performed using the Mann–Whitney test under the Bonferroni-corrected significance level (*p* < 0.017). ^a) b)^ Significant difference under pairwise post hoc test.

**Table 3:** Bone-to-implant contact and bone area fraction occupied in the three implant groups.

Parameter	Thread Implant (N = 7)	3D-Printed Implant without Spikes (N = 7)	3D-Printed Implant with Spikes (N = 8)	*p*-Value
BIC	52.27 ± 13.78	59.43 ± 16.98	44.28 ± 15.99	0.101
BAFO	56.79 ± 11.25	56.98 ± 12.48	45.58 ± 10.77	0.288

Values are presented as the mean ± standard deviation. BIC: bone-to-implant contact, BAFO: bone area fraction occupied. *p*-values were calculated using the Kruskal–Wallis test (*p* < 0.05).
